# Human Herpes Virus-6 (HHV-6) Reactivation after Hematopoietic Cell Transplant and Chimeric Antigen Receptor (CAR)- T Cell Therapy: A Shifting Landscape

**DOI:** 10.3390/v16040498

**Published:** 2024-03-24

**Authors:** Eleftheria Kampouri, Guy Handley, Joshua A. Hill

**Affiliations:** 1 Infectious Diseases Service, Lausanne University Hospital and University of Lausanne, CH-1011 Lausanne, Switzerland; 2 Department of Medicine, Division of Infectious Disease and International Medicine, Morsani College of Medicine, University of South Florida, Tampa, FL 33612, USA; guy.handley@moffitt.org; 3 H. Lee Moffitt Cancer Center and Research Institute, Tampa, FL 33612, USA; 4 Vaccine and Infectious Disease Division, Fred Hutchinson Cancer Center, Seattle, WA 98109, USA; jahill3@fredhutch.org; 5 Clinical Research Division, Fred Hutchinson Cancer Center, Seattle, WA 98109, USA; 6 Department of Medicine, University of Washington, Seattle, WA 98195, USA

**Keywords:** HHV-6, encephalitis, hematopoietic cell transplant, chimeric antigen receptor, CAR-T

## Abstract

HHV-6B reactivation affects approximately half of all allogeneic hematopoietic cell transplant (HCT) recipients. HHV-6B is the most frequent infectious cause of encephalitis following HCT and is associated with pleiotropic manifestations in this setting, including graft-versus-host disease, myelosuppression, pneumonitis, and CMV reactivation, although the causal link is not always clear. When the virus inserts its genome in chromosomes of germ cells, the chromosomally integrated form (ciHHV6) is inherited by offspring. The condition of ciHHV6 is characterized by the persistent detection of HHV-6 DNA, often confounding diagnosis of reactivation and disease—this has also been associated with adverse outcomes. Recent changes in clinical practice in the field of cellular therapies, including a wider use of post-HCT cyclophosphamide, the advent of letermovir for CMV prophylaxis, and the rapid expansion of novel cellular therapies require contemporary epidemiological studies to determine the pathogenic role and spectrum of disease of HHV-6B in the current era. Research into the epidemiology and clinical significance of HHV-6B in chimeric antigen receptor T cell (CAR-T cell) therapy recipients is in its infancy. No controlled trials have determined the optimal treatment for HHV-6B. Treatment is reserved for end-organ disease, and the choice of antiviral agent is influenced by expected toxicities. Virus-specific T cells may provide a novel, less toxic therapeutic modality but is more logistically challenging. Preventive strategies are hindered by the high toxicity of current antivirals. Ongoing study is needed to keep up with the evolving epidemiology and impact of HHV-6 in diverse and expanding immunocompromised patient populations.

## 1. Introduction

Human herpesvirus 6 (HHV-6) is a ubiquitous beta-herpesvirus that infects virtually all young children [1]. Primary infection is often asymptomatic or associated with a transient febrile illness, known as exanthema subitum, and leads to life-long latency with the potential to reactivate in the setting of immunosuppression [1]. HHV-6B is responsible for the majority of reactivation and disease, while the clinical significance of HHV-6A reactivation is less well understood and an association with disease is not well-established [2,3]. HHV-6B reactivation affects approximately half of all patients undergoing allogeneic hematopoietic cell transplant (HCT) [4,5]. While reactivation is usually asymptomatic, HHV-6B encephalitis can develop with high mortality and frequent long-term sequelae. HHV-6B has been associated with a plethora of clinical manifestations after HCT [4,6]. Chromosomally integrated HHV-6 (ciHHV6), a unique condition impacting approximately 1% of the human population, occurs when HHV-6 infects germ cells and is vertically transmitted to offspring. It is characterized by persistently high viral loads in blood and tissues and has been linked to adverse outcomes after HCT [7,8].

Our understanding of HHV-6B epidemiology is mostly based on historical studies in the allogeneic HCT setting, while a more contemporary perspective encompassing shifting clinical practices in HCT and expanding to novel cellular therapies such as chimeric antigen receptor (CAR)-T cell therapies is lacking. We herein review the epidemiology of HHV-6B after HCT, provide an update in the current era of post-transplant cyclophosphamide (PTCy) use for graft-versus-host disease (GVHD) prevention and letermovir for CMV prophylaxis, and expand to implications for CAR-T cell therapy recipients. We also review diagnostic, preventive, and therapeutic strategies and their challenges, highlighting gaps in knowledge and unmet needs to advance the field.

## 2. Epidemiology of HHV-6B after Allogeneic HCT

HHV-6A utilizes CD46 for cellular entry, a receptor regulating complement activation that is present on all nucleated cell lines. HHV-6B preferentially utilizes the CD134 receptor, part of the tumor necrosis factor receptor superfamily, expressed on activated T cells and associated with cytokines [9,10]. After infection, HHV-6A undergoes extensive histone modifications and appears to be transcriptionally silent [11]. No conclusive evidence implicates HHV-6A as a cause of disease following HCT [3]. HHV-6B demonstrates T cell tropism in vitro but infects various cell lines in vivo including various cells in the central nervous system (CNS), tonsils, salivary glands, kidney, liver, lungs, lymph nodes, and endothelium, in addition to monocytes, macrophages, and bone marrow progenitor cells [2,9].

### 2.1. Incidence, Viral Kinetics and Risk Factors for HHV-6B

HHV-6B reactivation affects approximately half of patients within the first 100 days after allogeneic HCT [5,12,13,14], while the incidence may exceed 90% in recipients of umbilical cord blood transplants or T cell depleted transplants [4,15,16]. HHV-6B is the most frequent infectious cause of encephalitis after allogeneic HCT [4,6,17,18,19]. Fortunately, HHV-6B encephalitis is still relatively rare, occurring in 1–3% of HCT recipients, though its incidence may be higher in specific groups (e.g., cord blood and T cell depleted transplants) [4,6,15]. Risk factors for reactivation and disease include umbilical cord blood transplant, T cell depleted allografts, human leukocyte antigen-mismatched or unrelated donors, acute GVHD and treatment with glucocorticoids [4,6,13,15,20].

HHV-6B reactivation usually occurs early after HCT, with a median time to reactivation of three weeks. In those who develop encephalitis, this usually follows quickly after viral detection in the blood [5,21]. A large study of allogeneic HCT recipients evaluated weekly plasma samples for the kinetics of multiple double stranded DNA viruses [21]. For HHV-6B, nearly half of all patients experienced a blip, defined as detection for ≤1 week, while less than a quarter of patients sustained HHV-6B detection in plasma for >4 weeks [21]. Notably, no patients with blips developed HHV-6B disease, and only 3.3% of 90 episodes of persistent HHV-6B detection were associated with HHV-6B end-organ disease [21]. Higher plasma viral loads (≥10,000 copies/mL) are frequently observed in patients who develop HHV-6B encephalitis [4,17]. In addition to the well-established link between higher HHV-6B plasma viral loads and encephalitis, a dose-dependent response between higher viral loads and mortality has also been reported [22].

### 2.2. An Update for the Current Clinical Practice Landscape

Major advances during the last decade and new practices in GVHD and infection prevention could impact the epidemiology of HHV-6B. PTCy for GVHD prophylaxis is more widely used in the setting of increasingly performed haploidentical and unrelated donor transplants [23]. PTCy is associated with increased risk for infections post-HCT in several studies [20,24,25,26,27]. A study of 2765 HCT recipients by the Center for International Blood and Marrow Transplantation Research showed an association between PTCy use and increased risk for non-CMV herpesvirus infection, primarily driven by HHV-6B, which was in turn associated with increased non-relapse mortality [24]. In another prospective study with weekly HHV-6 testing among 208 adult allogeneic HCT recipients, PTCy use was an independent risk factor for HHV-6 reactivation [20].

The advent of letermovir for CMV prophylaxis after allogeneic HCT marked a new era in CMV prevention [28]. Letermovir lacks activity against HHV-6B, and its use has led to a substantial decrease in the use of broad-spectrum antivirals for CMV [28,29,30]. Since these broad-spectrum antivirals have a prophylactic effect against HHV-6B, an increase in HHV-6B reactivation could be possible [21,31,32,33]. A large retrospective study among 738 HCT recipients compared the incidence of HHV-6B reactivation and disease (by clinical testing) in two cohorts: before and after the routine use of letermovir [34]. Reassuringly, the cumulative incidence of HHV-6B detection was similar in the two cohorts (3%), as was the incidence of HHV-6B encephalitis (0.5% and 0.6% pre- and post-letermovir, respectively), despite a decrease of approximately 40% in the use of broad-spectrum antivirals [34]. Some differences in viral kinetics were observed;HHV-6B detection shifted to earlier after HCT by a median of 10 days in the post-letermovir cohort [34]. Given that immune reconstitution improves with time after HCT, this earlier timeline could be associated with reduced immunological control [35]. Further, the peak plasma and CSF HHV-6B viral load tended to be higher by nearly 1 log_10_ copies/mL in the post-letermovir cohort compared to the pre-letermovir cohort [34]. Whether these changes are clinically relevant remains to be determined. These findings highlight the complexity of the effects of different practices on the epidemiology of HHV-6B and the need for large prospective studies with systematic testing to redefine HHV-6B epidemiology in the current era.

### 2.3. Clinical Manifestations and Diagnostic Pitfalls

The most established end-organ disease with a strong causal link to HHV-6B after HCT is encephalitis and may present with a well-defined post-transplant acute limbic encephalitis (PALE) syndrome with limbic system involvement [3,36,37,38]. Typical manifestations include acute CNS dysfunction including memory loss, altered mental status, and seizures. The detection of HHV-6B DNA in CSF coinciding with typical symptoms is considered diagnostic [3]. However, diagnosis of HHV-6B encephalitis is fraught with pitfalls; detection of HHV-6B DNA in CSF occurs in both asymptomatic patients as well as those with mental status changes due to alternative etiologies [39]. Alternative etiologies should be excluded prior to diagnosing HHV-6B encephalitis. High level DNAemia, defined as ≥10,000 copies/mL in plasma, has been previously shown to have relatively high specificity but poor sensitivity for the development of encephalitis or overall mortality based on historical data [4,17,38]. However, patient populations were often incompletely evaluated for HHV-6 DNA in CSF, and other studies have found probable cases of HHV-6B encephalitis absent high level DNAemia or even with only isolated CNS detection [32,39,40]. HHV-6 viral loads in the blood can rise rapidly around the time of symptom onset, which can limit the utility of serial PCR monitoring to preempt end-organ disease given the short window of opportunity to intervene [17,41]. Abnormal imaging or abnormal electroencephalogram findings are not always present in HHV-6B encephalitis and may develop after the onset of symptoms on repeat testing [36]. Additionally, initial PCR testing for HHV-6B DNA on CSF may be negative or below the level of detection in symptomatic patients and be found positive on subsequent evaluation [40]. Results may be discordant on different diagnostic testing platforms, then become concordant on subsequent testing, further highlighting the potential need for follow-up CSF evaluation in high-risk patients with consistent symptoms in the right context [40].

The wide cell tropism of HHV-6B in vivo translates into a wide spectrum of clinical manifestations, though the causality link is not always well-established [3]. Several epidemiologic associations of HHV-6B including fever and rash, GVHD [4,42], delayed engraftment and allograft dysfunction [13], hepatitis [43], and pneumonitis [22,44,45], have been proposed, though many are based on only weak or moderate evidence without a clear causal link [3,38]. In many instances, the detection of HHV-6B DNA reflects the net state of immunosuppression rather than the etiology of a given complication, although once the virus reactivates, it could further contribute to complications and worse outcomes. The detection of viral DNA in cellular samples is not diagnostic of end-organ disease as it can reflect infected lymphocytes or other latently infected cells [3]. When evaluating for HHV-6B disease, an extensive investigation to rule out alternative etiologies should be performed. 

There is accumulative evidence that HHV-6B reactivation is associated with, and potential playing some role in, the development of GVHD after HCT [42]. A systematic review and meta-analysis of the literature demonstrated that HHV-6B reactivation was significantly associated with increased risk for acute GVHD grades 2–4 (hazard ratio, 2.65; 95% confidence interval, 1.89–3.72; *p* < 0.001) [42]. Whether antiviral therapy for HHV-6B reactivation can impact the occurrence of acute GVHD remains unknown, and a potential mechanistic link has not been clearly demonstrated. However, this is an important area of study.

Emerging evidence suggests HHV-6B may be a causative pathogen of pneumonia after HCT [45]. In a large prospective study building off prior evidence, detection of HHV-6B DNA at a threshold of ≥2.8 log_10_ (≥578) copies/mL in bronchoalveolar fluid was highly correlated with detection of 2 HHV-6B mRNA transcripts indicative of lytic infection. Viral loads above this threshold were significantly associated with increased overall mortality and death due to respiratory failure in adjusted analyses [45]. Furthermore, distinct host gene expression signatures in patients with HHV-6B detection in bronchoalveolar lavage fluid further implicate HHV-6B as a distinct pathogen. These findings provide evidence to implicate HHV-6B as a pulmonary pathogen after allogeneic HCT, but whether treatment will improve outcomes has not been established. 

An association between HHV-6B and CMV reactivation has been reported both in allogeneic HCT and solid organ transplant recipients [5,46,47]. Even though the temporal association between the two viruses could simply reflect the state of severe immunosuppression, more complex mechanisms may be at play including evasion of virus-specific responses and viral persistence through the modification of the host-cell microenvironment, down regulation of HLA class I expression and synthesis of chemokine receptors [2,48]. The suppression of reconstitution of CMV-specific T cell-mediated immunity by HHV-6B has been reported as a potential mechanism [46], although data are conflicting [49].

### 2.4. Chromosomally Integrated HHV-6

**Figure 1:**
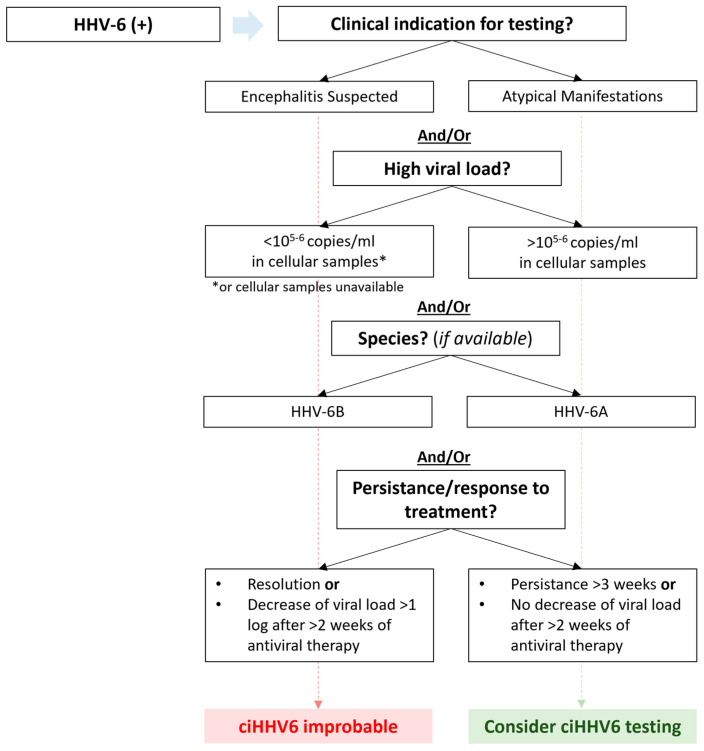
Algorithm for testing for chromosomally integrated HHV-6. Testing for chromosomally integrated HHV-6 (ciHHV6) should be considered in cases with atypical manifestations and/or high viral load > 10^5–6^ in cellular samples (assuming that white blood count is normal) and/or detection of species HHV-6A (based on data that HHV-6A reactivation and disease are rarely seen in this patient population and appear to be primary detected in ciHHV6). Testing should also be considered in the absence of a virological response despite antiviral treatment (e.g., persistent detection without a ≥log_10_ decline in viral load after ≥2 weeks). Testing of whole blood for ciHHV6 is available at University of Washington Virology Laboratory, Seattle, USA. (https://depts.washington.edu/uwviro/order/ accessed on 18 February 2024; https://testguide.labmed.uw.edu/view/HH6ABC?tabs=no accessed on 18 February 2024).

After primary infection in childhood, HHV-6A and HHV-6B can integrate their genome into telomeres of cell chromosomes [2,9]. Chromosomal integration may occur in germ cell lines leading to vertical transmission of ciHHV6 to half of the offspring through Mendelian inheritance, characterized by the presence of a copy of the HHV-6 genome in all nucleated cells. Inherited ciHHV6 occurs in 0.2–1% of all humans and leads to high levels of viral DNA in cellular samples including whole blood and tissue biopsies [2,7,50,51]. When HHV-6A is detected following HCT, ciHHV6 should always be considered, as HHV-6A reactivation is atypical but accounts for up to one third of cases of ciHHV6 [2,7,50,51]. The potential of the integrated virus for active replication and disease from an integrated state is controversial and requires further study, although cases have been well documented [3,50,52]. Importantly, ciHHV6 has been linked with adverse outcomes after HCT, including an increased risk for acute GVHD and CMV reactivation [7,8]. An algorithm for testing for ciHHV6 is provided in Figure 1, and testing methods are reviewed below.

## 3. Epidemiology of HHV-6B after Autologous HCT

The pathogenic role and epidemiology in the setting of autologous HCT is less understood. Autologous HCT recipients are regarded as being at lower risk for viral infection compared to allogeneic HCT [53,54]. A study with weekly multiplex PCR testing in blood for 13 viral pathogens, including most clinically relevant double-stranded DNA viruses, revealed detection HHV-6 in 42% of patients by the sixth week following autologous HCT [54]. HHV-6B reactivation is reported in 25–46% of autologous HCT recipients in small studies with weekly testing, and HHV-6B detection peaks at one to three weeks after HCT [54,55,56,57]. Retrospective studies reporting on clinically driven HHV-6B testing show an incidence of 9–16% [58,59,60]. Importantly, the main motive for clinical testing in these studies was unexplained peri-engraftment fever. Unexplained fever is the most common symptom reported at the time of HHV-6B detection in up to 90% of all events, followed by rash in 20–46% and lung infiltrates in 13–23%, while encephalitis is only sporadically reported [40,58,59]. However, fever of non-infectious cause develops frequently at engraftment after autologous HCT and is often attributed to engraftment syndrome. The non-specific nature of these manifestations and wide overlap with other causes (e.g., occult infection, drug reactions, auto-immunity) precludes establishing causal associations and highlights the need for validation in large cohorts.

## 4. Epidemiology of HHV-6B after CAR-T Cell Therapy

CAR-T cell therapy recipients often develop prolonged cytopenias and delayed T cell recovery and could be at risk for HHV-6B reactivation. The frequent use of corticosteroids for acute toxicities further impacts virus-specific cellular mediated immune responses [61,62,63]. Several cases of HHV-6B encephalitis and a case of fatal HHV-6B myelitis after CAR-T cell therapy have been reported [40,61,64,65,66,67,68,69]. However, the epidemiology and clinical significance of HHV-6B reactivation after CAR-T cell therapy are unknown. Overlapping clinical manifestations between viral encephalitis and immune effector cell-associated neurotoxicity syndrome (ICANS), occurring in 40–77% of CAR-T cell therapy recipients, makes diagnosis challenging without systematic testing [65,70]. Of note, routine surveillance in blood is typically not performed, and lumbar puncture and CSF analysis are infrequent after CAR-T cell therapy given the frequent clinical diagnosis of ICANS, precluding an accurate estimation of the incidence of HHV-6B reactivation and disease [71]. Finally, encephalitis represents probably only one facet, while atypical manifestations (delay in immune reconstitution, pneumonitis, fever, etc.) and other outcomes (myelosuppression, mortality, risk for infection) are also possible and could be overlooked in the setting of frequent febrile syndromes and late cytopenias attributed to immune effector cell-associated hematotoxicity [72].

In a study prospectively testing weekly blood samples for HHV-6 for 12 weeks after CD19 and BCMA CAR-T cell therapy, HHV-6 detection occurred in approximately 10% of participants between 2–6 weeks post-infusion [73]. Reactivation events were transient and low-level. In a large study of over 600 CAR-T cell therapy recipients, testing CSF for HHV-6B in the setting of ICANS was infrequent, however HHV-6B encephalitis appeared to be very infrequent with an estimated incidence of approximately 0.1% [71]. The potential role of cellular therapies as a source of viral infection in immunocompromised recipients was recently suggested [74]. The authors used large-scale viral genomics to examine the landscape of human latent viral reactivation and observed a signal for HHV-6 in T cell cultures in the absence of any record of HHV-6 in the original studies [74]. Zooming in on this finding, the authors used single-cell sequencing to identify a rare population of cells with high HHV-6 transcriptional activity in CD4+ T cells from in vitro cultures of pre-infusion CAR-T cells in addition to in vivo cultures from post-infusion blood. The conclusions of this study were that HHV-6 can reactivate in the process of generating CAR-T cell products, which could increase risk for transmission of an active viral infection to an immunosuppressed host [74]. These findings have important implications for manufacture of T cell therapeutics and monitoring of patients, but in the context of the results from the clinical studies discussed here, it appears that this may be of limited clinical significance.

Although there does not appear to be a role for routine monitoring of HHV-6B after CAR-T cell therapy, testing should be guided by clinical suspicion, particularly in cases of atypical ICANS or lack of response to standard treatments. Given that HHV-6B is a lymphotropic and neurotropic virus, its potential role in CAR-T cell therapy recipients with neurologic symptoms should be further explored. 

## 5. Diagnostic Strategies

Quantitative HHV-6B DNA assessment by real-time polymerase chain reaction (PCR) remains the primary diagnostic test while antigen or culture-based testing are rarely used [3]. Available PCR tests do not always discriminate between HHV-6A and HHV-6B, which is helpful when evaluating patients if feasible [3]. Inter-assay variability has been observed, though the development of a World Health Organization (WHO) international standard for HHV-6B PCR can facilitate comparability of results between studies [75,76]. The implementation of this new standard by major diagnostic labs will be a key step towards standardization and moving the field forward. Importantly, a positive PCR in tissue and other cellular samples can be difficult to interpret since it may reflect detection of latently infected cells. For this reason, cell-free plasma or serum are the preferred samples over whole blood when testing for HHV-6B reactivation and quantitative PCR provides important context compared to qualitative PCR [3,51]. 

The presence of high viral loads (often >10^5^–10^6^ copies/mL in cellular samples) and non-response to antiviral therapy are hallmarks of ciHHV6 [2,51]. Different constellations are seen based on whether ciHHV6 affects the donor, recipient, or both. In donor-derived ciHHV6, donor leukocytes carry the viral genome leading to high and persistent viral loads in hematopoietic tissues, including whole blood samples, after engraftment. In recipient ciHHV6, all nucleated non-hematopoietic cells carry the viral genome so detection in blood is variable and can reflect tissue damage and release of non-hematopoietic cellular genomes [3,77]. Of note, while extremely high viral loads are seen in cellular samples, viral loads tend to be 1–2 log_10_ lower in plasma and depend on timing of plasma processing from whole blood [51,78]. Testing of pre-HCT blood samples (or any sample in the donor) can help confirm ciHHV6 (especially when access to specific testing is limited). Droplet digital PCR to identify ciHHV6 allows for absolute quantification of targeted DNA and comparison of copies of viral genome to human cells (approximately 1 copy/cell is considered diagnostic), and though whole blood or other cellular samples are recommended, excellent specificity was reported for stored plasma samples [78,79,80]. In situ hybridization of telomeric integration sites is another assay than can be used for ciHHV6. These assays are not readily available in most laboratories and are not routinely performed in clinical practice. However, testing for ciHHV6 should be considered in select scenarios such as the absence of virologic response to antiviral therapy, persistent HHV-6 detection (e.g., ≥3 weeks) and/or detection of HHV-6A (Figure 1) [3,77].

## 6. Treatment Strategies

No controlled studies have demonstrated the optimal treatment strategy or duration of therapeutics for end-organ disease due to HHV-6B. Foscarnet, ganciclovir, and cidofovir have all demonstrated in vitro activity against the virus [81,82]. For HHV-6A, foscarnet and ganciclovir appear to be more effective in infected T cells, while foscarnet and cidofovir appear to be more effective for infected astroglioma cells in vitro [82]. For HHV-6B, all three drugs exhibit activity in T cell lines, but HHV-6B infection of astroglioma cells in vitro was shown to be abortive [82]. A study of 6593 allogeneic HCT patients, of which 145 developed HHV-6 encephalitis, showed a day +100 survival of 58.3% compared to 80.5% in those without the disease [6]. Of those with HHV-6 encephalitis, 57% had neurologic sequelae and nearly 80% were unable to return to work, school, or household activities, highlighting the potentially devastating nature of the disease and potential need for early intervention with therapeutics [6]. Receipt of a full dose of foscarnet of ≥180mg/kg/day or ganciclovir of ≥10 mg/kg/day was significantly associated with an improved neurologic response rate as well as lower rates of death or sequelae due to HHV-6 encephalitis compared to lower doses (56% vs. 75%; *p* = 0.022) [6]. This suggests that treatment with higher doses may mitigate end-organ disease. Receipt of foscarnet alone or in combination was associated with an improved 30-day overall survival, but no significant difference of ganciclovir monotherapy compared to foscarnet monotherapy [6]. While more investigation is needed, combination therapy potentially reduces neurologic sequelae but has not been shown to improve mortality [6,83]. 

**Figure 2:**
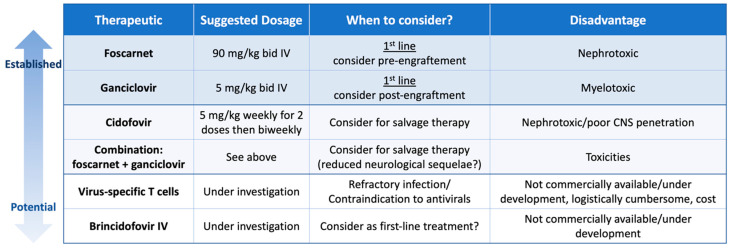
Therapeutics for HHV-6B. Intensity of color represents degree of evidence for use of each therapy, where darker color indicates more evidence. No treatments have an approval for HHV-6 except for foscarnet in Japan. Foscarnet and ganciclovir are first-line treatments, while cidofovir and combination treatment are reserved for salvage therapy. Virus-specific T cells and intravenous brincidofovir are investigational and not commercially available. Bid: twice per day; CNS: central nervous system; IV: intravenous.

There are three guidelines with recommendations for management of HHV-6B disease after HCT. The Japanese Society for Hematopoietic Cell Transplantation (JSHCT) focuses on HHV-6B encephalitis, the European Conference on Infections in Leukaemia (ECIL) gives comprehensive recommendations on evaluation and management of HHV-6B after HCT [3,84], and the American Society for Transplantation and Cellular Therapy provides some additional guidance for patients following cord blood transplant [85]. These guidelines recommend treatment with foscarnet 90 mg/kg bid or ganciclovir 5 mg/kg bid for at least 3 weeks and until documented clearance from blood, and if possible, clearance from the CSF. Combination therapy can be considered in severe cases [3,6,38,84]. Cidofovir may be considered as a salvage agent but is not generally recommended upfront due to concerns for poor CNS penetration [3,84]. Antiviral strategies are summarized in Figure 2.

Prophylactic and preemptive studies utilizing antivirals have unfortunately been unable to demonstrate clinical efficacy in preventing end-organ disease and can be associated with toxicity from antiviral agents [41,86,87,88,89]. One retrospective study of twenty-five umbilical cord blood transplant recipients receiving prophylactic foscarnet at a dose of 45 mg/kg twice a day, prophylaxis starting on day + 7 and continuing through engraftment, suggested a higher rate of neutrophil engraftment and overall survival at six months compared to a historical control [90]. The study was unable to show whether prophylaxis prevented either HHV-6B reactivation or disease due to its small size and lack of HHV-6 DNA monitoring in the historical control [90]. Another study evaluated preemptive foscarnet of 60–90 mg/kg/day for HHV-6 viremia in 11 patients but lacked a control group, limiting the ability to make claims of clinical efficacy [91]. Current guidelines recommend against preemptive and/or prophylactic therapy in routine practice [3,38,84].

Brincidofovir is a prodrug of cidofovir exhibiting high in vitro activity against HHV-6B. In a post-hoc analysis of a controlled trial of twice-weekly oral brincidofovir for CMV prophylaxis after HCT, brincidofovir led to a lower incidence of HHV-6B reactivation (14% in the treatment group and 32% in the placebo (*p* = 0.019)) [33]. Furthermore, brincidofovir was associated with a lower viral load among those with reactivation [33]. The intravenous preparation of brincidofovir lacks the gastrointestinal toxicity of the oral form and is under development. These encouraging data highlight a potential role for prophylactic strategies if safe and well-tolerated antiviral molecules, or other strategies (e.g., virus-specific T cells), become available. 

## 7. Virus-Specific T cells

Adoptive immunotherapy with virus-specific T cells is a promising therapeutic approach that could change the paradigm in the treatment and prevention of double-stranded DNA viral infections in immunocompromised hosts. Such products have been studied for treatment, preemptive therapy, and prophylaxis [92,93]. Phase 1/2 trials of off-the-shelf, virus-specific, or multivirus-specific products have shown safety and potential efficacy, but phase 3 trials with a control arm are lacking [92]. In a small study without a control group, the use of HHV-6B specific T cells appeared to be effective in the treatment of refractory HHV-6B reactivation after allogeneic HCT, including a case of HHV-6B encephalitis not responding to antiviral therapy [94]. Similar results were observed in a phase 2 study of an off-the-shelf multivirus-specific product [95]. The use of an allogeneic multivirus-specific T cell product administered every two weeks after engraftment for up to seven infusions as prophylaxis for double stranded DNA viruses, including HHV-6, was associated with a lower-than-expected rate of clinically significant viral infection in a phase 2 study among 26 high-risk allogeneic HCT recipients [96]. Unfortunately, a recently conducted phase 3 randomized controlled trial was terminated based on a pre-specified futility assessment, although HHV-6 was not a primary endpoint [97]. Importantly there were no safety concerns, however the results are not yet available.

## 8. Discussion and Future Directions

HHV-6B remains an intriguing if not elusive viral infection that is frequently encountered by health care providers caring for cellular therapy recipients. A few decades have passed since HHV-6 was first identified, yet HHV-6 is still shrouded by uncertainty and speculation [98]. Its ubiquitous nature, wide cell tropism, unique latency mechanism and potential for vertical transmission to offspring all add to its allure. Improved diagnostic methods have helped better understand the epidemiology and the pathogenic role, but contemporary epidemiological studies reflecting current clinical practices in HCT and encompassing novel cellular immunotherapies are lacking. The development of a WHO standard for HHV-6 PCR can increase comparability between studies if used, but heterogeneity in diagnostic methods are ongoing hurdles for standardization and comparability of studies. Further, broader availability of diagnostic assays for ciHHV6 is a prerequisite to better understand the role and Impact of this condition on patient management and outcomes. 

Despite uncertainty regarding the optimal treatment, the choice of antiviral is limited to the few currently available agents and mostly influenced by the toxicity profile. Novel approaches including adoptive therapy with virus-specific T cells bear further development. Mounting evidence shows that preemptive therapy of asymptomatic HHV-6B DNAemia after HCT is of limited utility with currently available antivirals, and treatment should be reserved for end-organ disease in the context of our current treatment arsenal wherein the toxicities may mitigate the potential benefits. Following the paradigm of letermovir for CMV prophylaxis, the availability of safer therapeutics would renew the interest in preventive strategies and potentially justify routine monitoring. However, to successfully implement preventive strategies, further evidence is required to understand the clinical spectrum of disease in various emerging populations and determine those who may derive the most benefit.

## 9. Conclusions

The epidemiology of HHV-6B needs to be further elucidated in the current era to reflect contemporary HCT practices and a rapidly evolving patient population treated with novel cellular therapies. Beyond the causal link of HHV-6B with encephalitis after HCT, its clinical impact is incompletely understood and overlaps with other syndromes following HCT. Understanding the full spectrum of HHV-6 reactivation and disease, as well as the pathogenic role of ciHHV6, will require standardized and more readily available diagnostic assays. Advances in diagnostic methods and the advent of safer antiviral agents may facilitate risk-adapted approaches to prophylactic and preemptive treatment strategies.
